# Supplementary material for: The Moderating Role of Emotional Intelligence on the Relationship Between Nurses’ Preparedness to Care for COVID-19 Patients and Their Quality of Work Life
Source: Behav Sci (Basel). 2024 Dec 5;14(12):1166. doi: 10.3390/bs14121166 (PMC11672974; doi:10.3390/bs14121166)
Supplement: Supplementary file 1 [file behavsci-14-01166-s001.zip › behavsci-3299708-supplementary/supplementary_S1.pdf]

S1: Descriptive table for the subscales' items of Emotional Intelligence among nurses working during the COVID-19 pandemic (N = 267).

**Self-Awareness**	**Strongly Agree**	**Agree**	**Neutral**	**Disagree**	**Strongly Disagree**
I understand the relationship between my feelings and what I think, do and say.	47 (17.6)	157 (58.8)	55 (20.6)	5 (1.9)	3 (1.1)
I recognise how my feelings affect my performance	48 (18)	181 (67.8)	27 (10.1)	9 (3.4)	2 (0.7)
I am aware of my goals and values	67 (25.1)	172 (64.4)	21 (7.9)	4 (1.5)	3 (1.1)
I am aware of my strengths and weaknesses	77 (28.8)	161 (60.3)	24 (9)	3 (1.1)	2 (0.7)
I try to learn from experiences	90 (33.7)	157 (58.8)	15 (5.6)	3 (1.1)	2 (0.7)
I am open to continuous learning, self development, new perspectives &honest feedback	85 (32)	161 (60.5)	15 (5.6)	3 (1.1)	2).7)
I am able to show sense of humour and perspective about myself	65 (24.2)	163 (61)	33 (12.4)	2 (0.7)	4 (1.5)
I present myself with self-assurance; I have "presence"	60 (22.5)	171 (64)	32 (12)	3 (1.1)	1 (0.4)
I am organized and careful in my work	88 (33)	148 (55.4)	27 (10.1)	2 (0.7)	2 (0.7)
I usually go for original ideas while solving a problem	61 (22.8)	166 (62.2)	35 (13.1)	4 (1.5)	1 (0.4)
I am able to make sound decisions despite uncertainties and pressures	42 (15.7)	173 (64.8)	41 (15.4)	8 (3)	3 (1.1)
**Emotional regulation**	**Strongly Agree**	**Agree**	**Neutral**	**Disagree**	**Strongly Disagree**
I usually feel depressed for one reason or the others	17 (6.4)	93 (34.8)	95 (35.6)	49 (18/4)	13 (4.9)
I feel happy and satisfied about my life	64 (24.6)	149 (52.6)	50 (18.8)	9 (3.4)	3 (1.1)
I can predict clearly whether my emotion is happy or sad	43 (16.1)	157 (58.8)	58 (21)	6 (2.2)	5 (1.9)
I am someone who is original and don't copy others	68 (25.5)	158 (58.4)	33 (12.4)	4 (1.5)	6 (2.2)
I am quite a cheerful and lively person	63 (23.6)	153 (57.3)	44 (16.5)	4 (1.5)	3 (1.1)

I can win over stress without getting too nervous	29 (10.9)	144 (53.9)	70 (26.2)	16 (6)	8 (3)
I manage my impulsive feelings and disappointing emotions well	30 (11.2)	169 (63.3)	62 (23.2)	4 (1.5)	2 (0.7)
I keep myself positive, composed and calm even in frustrating situations	39 (14.6)	174 (65.2)	47 (17.6)	4 (1.5)	3 (1.1)
I think clearly and stay focused under pressure	41 (15.4)	178 (66.7)	41 (15.4)	5 (1.9)	2 (0.7)
I know how to keep myself calm in conflicting and upsetting problems	37 (13.9)	170 (63.7)	49 (18.4)	7 (2.6)	4 (1.5)
I get carried away with my imagination and daydreaming most often	8 (3)	111 (41.6)	58 (21.7)	75 (28.1)	15 (5.6)
I feel cool, relaxed& stress free most of the times	15 (5.6)	128 (47.9)	84 (31.5)	34 (12.7)	6 (2.2)
When I hear bad news, I usually can't control myself and feel sad and miserable	6 (2.2)	95 (35.6)	89 (33.3)	68 (25.5)	9 (3.4)
I can avoid external temptations in order to fulfil my dream	21 (7.9)	161 (60.3)	68 (25.5)	11 (4.1)	6 (2.2)
I smoothly handle multiple demands, shifting priorities and rapid change	16 (6)	159 (59.6)	77 (28.8)	8 (3)	7 (2.6)
**Self Motivation**	**Strongly Agree**	**Agree**	**Neutral**	**Disagree**	**Strongly Disagree**
I am result-oriented with a high drive to meet objectives and goals	28 (10.5)	171 (64)	59 (22.1)	4 (1.5)	5 (1.9)
I continuously learn in order to improve my performance	52 (19.5)	173 (64.8)	34 (12.7)	4 (1.5)	4 (1.5)
Before beginning something new, I usually feel that I will succeed.	30 (11.2)	169 (63.3)	61 (22.8)	4 (1.5)	3 (1.1)
I pursue goals beyond what's required or expected of me.	24 (9)	175(65.5)	49 (18.4)	4 (1.5)	1 (0.4)
I am determined in achieving goals despite obstacles and setbacks	38 (14.2)	175 (65.5)	49 (18.4)	4 (1.5)	1 (0.4)
I possess good confidence in taking sole responsibility and taking decisions by my own	36 (13.5)	170 (63.7)	54 (20.2)	5 (1.9)	2 (0.7)

I hold myself accountable for meeting my objectives	42 (15.7)	170 (63.7)	48 (18)	6 (2.2)	1 (0.4)
When working in team, I like to depend upon other's ideas than on my own	10 (3.7)	105 (39.3)	98 (36.7)	47 (17.6)	7 (2.6)
I am generally motivated to continue, even when situations become worse to handle	21 (7.9)	182 (68.2)	58 (21.7)	4 (1.5)	2 (0.7)
**Social Awareness**	**Strongly Agree**	**Agree**	**Neutral**	**Disagree**	**Strongly Disagree**
I understand the way others think feel and behave.	32 (12)	174 (65.2)	52 (19.5)	5 (1.9)	4 (1.5)
People think that I am optimistic and self-confident person.	24 (9)	155 (58.1)	75 (28.1)	10 (3.7)	3 (1.1)
Others think that I lack confidence in interacting with others.	8 (3)	76 (28.5)	74 (27.7)	92 (34.5)	17 (6.4)
I show sensitivity and understand others' point of view	29 (10.9)	170 (63.7)	54 (20.2)	11 (4.1)	3 (1.1)
I recognise and reward people's strengths, accomplishment and development	37 (13.9)	176 (65.9)	50 (18.7)	3 (1.1)	1 (0.4)
I respect and relate well to people from different backgrounds.	53 (19.9)	174 (65.2)	36 (13.5)	2 (0.7)	2 (0.7)
I see variety in people as opportunity, creating an environment where diverse people can prosper.	35 (13.1)	169 (63.3)	56 (21)	4 (1.5)	3 (1.1)
It's quite easy for me to understand the non-verbal messages (facial expressions) of others.	33 (12.4)	158 (59.2)	61 (22.8)	13 (4.9)	2 (0.7)
I can tell how others are feeling by listening to their tone of voice	35 (13.1)	170 (63.7)	51 (19.1)	9 (3.4)	2 (0.7)
**Social Skills**	**Strongly Agree**	**Agree**	**Neutral**	**Disagree**	**Strongly Disagree**
I am skilled at the art of convincing others	22 (8.2)	134 (50.2)	95 (35.6)	15 (5.6)	1 (0.4)
I am easy to get friendly and possess good social skills.	40 (15)	164 (61.4)	54 (20.2)	7 (2.6)	2 (0.7)

I promote open communication and ready to accept both bad and good news	41 (15.4)	176 (65.9)	45 (16.9)	4 (1.5)	1 (0.4)
I am extremely polite &respectful to others irrespective of the unfavourable circumstances	41 (15.4)	157 (58.8)	65 (24.3)	2 (0.7)	2 (0.7)
I handle difficult people and tense situations with diplomacy and tact.	27 (10.1)	169 (63.3)	63 (23.6)	6 (2.2)	2 (0.7)
I encourage open discussion and debate	29 (10.9)	153 (57.3)	73 (27.3)	10 (3.7)	2 (0.7)
I look forward to relationships that are mutually useful.	33 (12.4)	174 (65.2)	49 (18.4)	9 (3.4)	2 (0.7)
I keep others in a team and build a strong bond	47 (17.6)	176 (65.9)	40 (15)	3 (1.1)	1 (0.4)
I make and maintain personal friendships among work associates.	37 (13.9)	164 (61.4)	56 (21)	6 (2.2)	4 (1.5)
I maintain a balance between work and relationships.	42 (15.7)	180 (67.4)	37 (13.9)	4 (1.5)	4 (1.5)
I promote a friendly and cooperative climate	47 (17.6)	176 (65.9)	39 (14.6)	3 (1.1)	2 (0.7)
I look for opportunities to work in a team	43 (16.1)	185 (69.3)	34 (12.7)	3 (1.1)	2 (0.7)
I find it difficult to get friendly with someone who is not known to me	12 (4.5)	106 (39.7)	100 (37.5)	43 (16.1)	6 (2.2)
**Emotional Repetitive**	**Strongly Agree**	**Agree**	**Neutral**	**Disagree**	**Strongly Disagree**
I like to cooperate with others in accomplishing a task.	48 (18)	179 (67)	35 (13.1)	1 (0.4)	4 (1.5)
I help others in coming out of difficult situations	42 (15.7)	176 (65.9)	44 (16.5)	3 (1.1)	2 (0.7)
I extend support and advice to others when needed.	39 (14.5)	180 (67.4)	43 (16.1)	3 (1.1)	2 (0.7)
Others find it comfortable to disclose their personal problems	21 (7.9)	143 (53.6)	88 (33)	11 (4.1)	4 (1.5)
I help other people feel better when they are in bad mood	31 (11.6)	171 (64)	60 (22.5)	1 (0.4)	4 (1.5)
I offer useful feedback and identify people's needs for development	29 (10.9)	175 (65.5)	57 (21.3)	2 (0.7)	4 (1.5)
I listen well, seek mutual understanding and fully	41 (15.4)	175 (65.5)	46 (17.2)	3 (1.1)	2 (0.7)

welcome sharing of information					
I guide the performance of others while holding them accountable	25 (9.4)	159 (59.6)	72 (27)	8 (3)	3 (1.1)
I am more of a leader than a follower	16 (6)	116 (43.4)	105 (39.3)	23 (8.6)	7 (2.6)
I act as a mediator in resolving conflict between two parties	19 (7.1)	139 (52.1)	99 (37.1)	9 (3.4)	1 (0.4)
I can easily detect the differences between others' feeling and behaviors	20 (7.5)	164 (61.4)	74 (27.7)	6 (2.2)	3 (1.1)
Its not easy for me to accurately relict people's feeling back to them.	8 (3)	110 (41.2)	116 (43.4)	25 (9.4)	8 (3)
