# Supplementary material for: The Moderating Role of Emotional Intelligence on the Relationship Between Nurses’ Preparedness to Care for COVID-19 Patients and Their Quality of Work Life
Source: Behav Sci (Basel). 2024 Dec 5;14(12):1166. doi: 10.3390/bs14121166 (PMC11672974; doi:10.3390/bs14121166)
Supplement: Supplementary file 1 [file behavsci-14-01166-s001.zip › behavsci-3299708-supplementary/supplementary_S2.pdf]

S2: Descriptive table for the subscales' items of Quality of Work Life among nurses working during the COVID-19 pandemic (N = 267).

Quality of Work Life	Response				
	Strongly Agree	Agree	Neutral	Disagree	Strongly Disagree
**I. Homelife/Work life**					
I am able to balance work with my family needs	12 (4.5)	120 (44.9)	60 (22.5)	48 (18)	27 (10.1)
It is important for the hospital to offer employee on-site children service	47 (17.6)	167 (62.5)	41 (15.4)	5 (1.9)	7 (2.6)
I have energy left after work.	9 (3.4)	66 (24.7)	57 (21.3)	89 (33.3)	46 (17.2)
Rotating schedules negatively affect my life.	8 (3)	43 (16.1)	69 (25.8)	100 (37.5)	47 (17.6)
My organization's policy for family-leave time is adequate	2 (0,7)	93 (34.8)	75 (28.1)	63 (23.6)	34 (12.7)
It is important for a hospital to offer employees on-site day care for elderly parents	25 (9.4)	157 (58.8)	63 (23.6)	18 (6.7)	4 (1.5)
It is important for a hospital to offer employees on-site ill child care services.	37 (13.9)	171 (64)	43 (16.1)	11 (4.1)	5 (1.9)
**II. Work Design**					
I receive a sufficient amount of assistance from unlicensed support personnel (the dietary aides, housekeeping, patient care technicians, and nursing assistants).	12 (4.5)	120 (44.9)	60 (22.5)	48 (18)	27 (10.1)
I am satisfied with my job.	38 (14.2)	158 (59.2)	43 (16.1)	12 (4.5)	16 (6)
My workload is too heavy.	56 (21)	121 (45.3)	56 (21)	22 (8.2)	12 (4.5)
I have the autonomy to make patient care decisions.	16 (6)	155 (58.1)	68 (25.3)	20 (7.5)	8 (3)

I perform many non-nursing tasks.	65 (24.4)	105 (39.5)	55 (20.7)	32 (12)	9 (3.4)
I experience many interruptions in my daily work routine.	23 (8.6)	130 (48.7)	72 (27)	34 (12.7)	8 (3)
I have enough time to do my job well.	12 (4.5)	120 (44.9)	75 (28.1)	46 (17.2)	14 (5.2)
I am able to provide good quality patient care.	72 (27)	156 (58.4)	23 (8.6)	10 (3.7)	6 (2.2)
I receive quality assistance from unlicensed support personnel (the dietary aides, housekeeping, patient care technicians, and nursing assistants).	10 (3.7)	151 (56.6)	67 (25.1)	30 (11.2)	9 (3.4)
**III. Work Context**					
I am able to communicate well with my nurse manager/supervisor	21 (7.9)	131 (49.1)	57 (21.3)	35 (13.1)	23 (8.6)
I have adequate patient care supplies and equipment.	16 (6)	155 (58.1)	68 (25.5)	20 (7.5)	8 (3)
My nurse manager/supervisor provides adequate supervision	35 (13.1)	173 (64.8)	34 (12.7)	10 (3.7)	15 (5.6)
Friendships with my co-workers are important to me.	78 (29.2)	164 (61.4)	14 (5.2)	3 (1.1)	8 (3)
My work setting provides career advancement opportunities.	16 (6)	152 (56.9)	58 (21.7)	30 (11.2)	11 (4.1)
There is teamwork in my work setting	36 (13.5)	185 (69.3)	31 (11.6)	11 (4.1)	4 (1.5)
I feel a sense of belonging in my workplace.	14 (5.2)	156 (58.4)	66 (24.7)	16 (6)	15 (5.6)
I am able to communicate with the other therapists (physical, respiratory, etc.).	26 (9.7)	171 (64)	49 (18.4)	13 (4.9)	8 (3)
I receive feedback on my performance from my	17 (6.4)	176 (65.9)	46 (17.2)	17 (6.4)	11 (4.1)

nurse manager/supervisor.					
I am able to participate in decisions made by my nurse manager/supervisor	13 (4.9)	135 (50.6)	66 (24.7)	40 (15)	13 (4.9)
I feel respected by physicians in my work setting.	33 (12.4)	162 (60.7)	52 (19.5)	15 (5.6)	5 (1.9)
It is important to have a designated, private break area for the nursing staff.	100 (37.6)	131 (49.1)	24 (9)	7 (2.6)	5 (1.9)
It is important to me to have nursing degree-granting programs available at my hospital.	52 (19.5)	177 (66.3)	29 (10.9)	6 (2.2)	3 (1.1)
I receive support to attend in-services and continuing education programs.	22 (8.2)	161 (60.3)	52 (19.5)	21 (7.9)	11 (4.1)
I communicate well with the physicians in my work setting.	41 (15.4)	190 (71.2)	23 (8.6)	9 (3.4)	4 (1.5)
I am recognized for my accomplishments by my nurse manager/supervisor.	13 (4.9)	150 (56.2)	70 (26.2)	21 (7.9)	13 (4.9)
Nursing policies and procedures facilitate my work.	38 (14.2)	175 (65.5)	41 (15.4)	7 (2.6)	6 (2.2)
The security department provides a secure environment.	20 (7.5)	155 (58.1)	59 (22.1)	22 (8.2)	11 (4.1)
I feel safe from personal harm (physical, emotional, or verbal) at work.	20 (7.5)	125 (46.8)	71 (26.6)	34 (12.7)	17 (6.4)
Upper-level management has respect for nursing	16 (6)	129 (48.3)	74 (27.7)	26 (9.7)	22 (8.2)
**IV. Work world**					
In general, society has an accurate image of nurses.	38 (14.2)	158 (59.2)	43 (16.1)	12 (4.5)	16 (6)
My salary is adequate for my job given the current job market conditions.	3 (1,1)	91 (34.1)	55 (20.6)	59 (22.1)	59 (22.1)
I would be able to find my same job in another	35 (13.1)	114 (42.7)	77 (28.8)	29 (10.9)	12 (4.5)

organization with about the same salary and benefits.					
I believe my job is secure.	18 (6.7)	124 (46.4)	80 (30)	28 (10.5)	17 (6.4)
My work impacts the lives of patients/families.	51 (19.1)	164 (61.4)	37 (13.9)	10 (3.7)	5 (1.9)
